# In Vitro Antioxidant, Cytotoxic Activities, and Phenolic Profile of *Senecio glaucus* from Saudi Arabia

**DOI:** 10.1155/2020/8875430

**Published:** 2020-10-24

**Authors:** Ali S. Alqahtani, Rashed N. Herqash, Omar M. Noman, Fahd A. Nasr, Nouf Alyhya, Shamsa H. Anazi, Muhammad Farooq, Riaz Ullah

**Affiliations:** ^**1**^ Department of Pharmacognosy, College of Pharmacy, King Saud University, P.O. Box 2457, Riyadh 11451, Saudi Arabia; ^2^Medicinal, Aromatic and Poisonous Plants Research Center, College of Pharmacy, King Saud University, Riyadh 11451, Saudi Arabia; ^3^Department of Zoology, College of Science, King Saud University, P.O. Box 2455, Riyadh 11451, Saudi Arabia

## Abstract

Current treatments for complex diseases have remarkable side effects that negatively impact patients' quality of life. Thus, natural compounds with fewer side effects represent a promising source for safe drugs. The genus *Senecio* is widely used in folk medicine due to its various pharmacological properties. In the present study, the total phenolic content of *Senecio glaucus*, which is grown in Saudi Arabia, was assessed using the Folin-Ciocalteau colorimetric method. Scavenging DPPH and ABTS assays were utilized to determine the antioxidant properties of *S. glaucus* fractions, and MTT assay was used to screen the cytotoxic activity of *S. glaucus* against various cancer cells. In addition, HPLC-UV was utilized to detect the presence of two phenolic acids, namely, vanillic acid (VA) and gallic acid (GA). Among all fractions tested, *S. glaucus* chloroform fraction (SGCF) yielded the highest value (125.3 mg·GA/g) in terms of total phenolic content. SGCF also exhibited the highest scavenging activities (76.7 and 74.1%) on both DPPH and ABTS assays, respectively. Similarly, SGCF also possessed the most potent cytotoxic activity against the MCF-7 cell line, with an IC_50_ value of 41.8 *μ*g/ml. The validated HPLC method confirmed the presence of VA (4.8 *μ*g/mg DW) and GA (3.9 *μ*g/mg DW) in SGCF. Overall, our data show that *S. glaucus* had antioxidant and cytotoxic properties. A developed validated HPLC method which could be helpful for quantifying phenolic compounds in *S. glaucus* was established.

## 1. Introduction

Medicinal plants have been widely explored for their various biological activities and due to their safety and efficacy [1]. Phenolic compounds are a type of phytocompounds that are ubiquitously found in several plant species. These compounds have been widely studied due to their enormous beneficial properties and biological activities [2]. Additionally, several plants rich in phenolic compounds have shown promising results for reducing or preventing various chronic diseases, including cancer and cardiovascular diseases [3]. Furthermore, the phenolic content in these plants allows them to have several beneficial properties, including antioxidant and anticancer properties [4]. Therefore, the potential antioxidant and anticancer properties from new plant species are being studied on a global scale.

The *Senecio* genu*s* represents the largest genus of the Asteraceae family and includes more than 1500 species of herbs and trees [5]. *Senecio* species have traditionally been utilized in folk medication for various ailments, including treatment for cough, accelerating wound healing, and as treatments for asthma and eczema [6]. In addition, several classes of various natural compounds from the *Senecio* genera have been isolated and characterized [7–10]. Some of these classes of natural products have shown significant medicinal properties, such as antiviral [11], antioxidant [12], antifungal, antibacterial [13], antidiabetic [14], and cytotoxic properties [15–17].

*Senecio glaucus* is one of the most important species from the *Senecio* genus. It is an annual herbaceous plant. The presence of bioactive compounds in *S. glaucus*, such as phenolics, saponins, flavonoids, and tannins, as well as some volatile essential oils, has been reported [18]. *S. glaucus* reportedly exhibits significant cytotoxic activity against colon carcinoma cells [19]. As part of our systematic investigation of various plant species scattered in Saudi Arabia, we aimed to evaluate the in vitro antioxidant and cytotoxic effects of *S. glaucus*. In addition, we also aimed to quantitatively estimate the presence of total phenolics and some phenolic acids, such as gallic acid (GA) and vanillic acid (VA).

## 2. Materials and Methods

### 2.1. Plant Collection and Authentication

The aerial parts (leaves and stems) from *S. glaucus* were collected during the autumn season in 2018 from the southern region of Saudi Arabia. To ensure that the leaves and stems collected were *S. glaucus*, these were authenticated by an expert taxonomist in the herbarium of Pharmacognosy Department, College of Pharmacy, King Saud University, Riyadh, Saudi Arabia.

### 2.2. Crude Extracts and Fractions Preparation

The aerial parts of *S. glaucus* were dried in a ventilated room and crushed to a fine powder. The crude extract was prepared by soaking 500 g of the dry powder in 3 L of ethanol for 48 h. A rotary evaporator was then used to concentrate the crude extract (60 g) which was then partitioned using different solvents, including *n-*hexane (Hex), chloroform (CHCl_3_), and *n-*butanol (ButOH) producing 8 g, 13 g, and 22 g of dried extracts, respectively.

### 2.3. Estimation of Total Phenol Content

The crude extract and fractions of *S. glaucus* were quantitatively examined for total phenolic content using the standard Folin-Ciocalteau spectrophotometric method [20]. For this procedure, 0.5 ml of each sample was added to 0.1 ml of Folin-Ciocalteau reagent (0.5 N) and mixed thoroughly. Subsequently, 2.5 ml of sodium carbonate (Na_2_CO_3_) was added, and the mixture was left to stand for 0.5 h after mixing. Optical density was measured at 760 nm. Total phenolic contents were presented as milligram equivalents of GA (GAE)/g of the extract.

### 2.4. Determination of Antioxidant Activity

#### 2.4.1. DPPH Radical Scavenging Activity

The crude extract and fractions of *S. glaucus* were screened for antioxidative activity using 2,2-diphenyl-1-picrylhydrazyl (DPPH) as described by Brand et al. [21]. In brief, 500 *μ*L of each sample at various concentrations from each fraction was mixed with 125 *μ*L DPPH solution (1 mM) and 375 *μ*L methanol and further incubated for 30 min. Finally, UV-spectrophotometer absorbance measurement at *λ* = 517 nm was applied to demonstrate the anti-DPPH activity using the following formula:

(1) $$\begin{array}{c} \%\text{ of antiradical activity}=\frac{\text{Abs of control}-\text{Abs of sample}}{\text{Abs of control}\times 100}. \end{array}$$

#### 2.4.2. ABTS Radical Cation Scavenging Activity

The antioxidant capacity of *S. glaucus* crude extract and fractions was estimated utilizing the ABTS method as utilized by Li et al. [22], with slight modifications. In brief, ABTS (7 mM) and potassium persulfate (2.45 mM) aqueous solutions were mixed, incubated for 0.5 h, and kept in the refrigerator for 24 h, after which it was diluted with ethanol. Thereafter, the ABTS solution (50 *μ*g/ml) was mixed with different concentrations for each fraction (1 : 1). The antioxidant percentage activity of *S. glaucus* fractions was demonstrated based on the reduction of ABTS, which was optically measured at 734 nm using the following formula [23]:

(2) $$\begin{array}{c} \%\text{ of radical scavenging activity}=\frac{\text{Abs control}-\text{Abs sample}}{\text{Abs control}\times 100.} \end{array}$$

### 2.5. Cell Viability (MTT Assay)

Cell viability MTT assay was carried out according to Alqahtani et al. [24]. In brief, A549 (lung), MCF-7 (breast), and HepG2 (liver) cancer cells were harvested, counted, and plated on the bottom of a 24-well plate (5 x  10^4^ cells/well) for 24 h to allow for attachment. Various concentrations of *S. glaucus* fractions were added to each well and incubated with the cells at 37°C in the presence of 5% CO_2_. After 48 h, 0.1 ml of 5 mg/ml MTT was added to each well, and the plates were again incubated at 37°C and 5% CO_2_ for 2–4 hours. Next, the media were aspirated, a solution of 0.01 N HCl in isopropanol was added to each well, and the plates were placed in a shaker for 10 min to dissolve the formed formazan crystals. The absorbance was read at 570 nm using a microplate reader. The concentration at which cell viability is reduced by 50% (IC_50_) was calculated using OriginPro 8.5 software.

### 2.6. HPLC Analysis

HPLC-grade acetonitrile and acetic acid were obtained from VWR International Ltd. (Poole, England). The water used in the analysis was purified by a Milli-Q purification system manufactured by Purelab Flex: Elga Veolia Ltd. (High Wycombe, UK). Standard compounds of gallic acid (≥96%) and vanillic acid (99%) were purchased from Sigma Aldrich (St. Louis, MO, USA). All solvents used during the mobile phase were previously filtered through 0.45 *μ*m Millipore and degassed prior to use. The standard solutions were prepared by dissolving the accurate weights of the reference standards in methanol to a concentration of 0.5 mg/mL and then further diluted with additional methanol to arrive at different diluted solutions for the preparation of standard calibration curves. All samples for HPLC analysis were filtered through 0.45 *μ*m Millipore and immediately used for analysis.

#### 2.6.1. Apparatus and Operating Parameters

The analysis was carried out by Shimadzu chromatographic system (Shimadzu, Kyoto, Japan) equipped with binary solvent delivery units (LC-10AD Prominence liquid chromatography pumps), Prominence UV/Vis detector (SPD-20A), and a system controller (CBM-20A Prominence Communication Bus Module) with LC solution software. Chromatographic analyses were performed on Shim-pack VP-ODS C18 reversed-phase column (250 × 4.6 mm, 5 *μ*m, Shimadzu, Kyoto, Japan). The mobile phase was acetonitrile–water–acetic acid (20 : 80 : 1) and increasing up to acetonitrile–water–acetic acid (80 : 20 : 1) in 30 minutes. Chromatographic operations were carried out at a constant flow rate (1 ml/min) and ambient temperature. Detection was performed at 277 nm according to the ultraviolet (UV) absorption maxima of the two analytes.

### 2.7. Statistical Analysis

Each assay was done in triplicate for each fraction. The data presents the mean ± SD from three independent experiments.

## 3. Results

### 3.1. Phenolic Contents of *S. glaucus*

**Figure 1:**
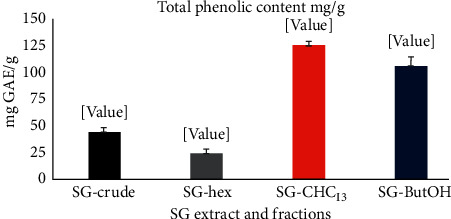
*S. glaucus* total phenolic content.

The total phenolic contents from crude and fractions of *S. glaucus* are presented in Figure 1. The results show that the chloroform fraction had the highest concentration of phenolic contents (125.3 mg·GA/g). The remaining fractions also showed varied contents in terms of mg·GA/g of dry extract (Figure 1).

### 3.2. Antioxidant Activity

The radical scavenging activity of *S. glaucus* fractions is shown in Table 1. In the ABTS test, *S. glaucus* displayed a remarkable capacity to scavenge free radicals at various concentrations. It was found that SGCF had the highest antioxidant content at a concentration of 1000 *μ*g/mL with a value of 76.7 and 74.1% in DPPH and ABTS, respectively. In addition, the remaining fractions also showed variable antioxidant activities in both tests (Table 1).

### 3.3. Cytotoxicity of Different Cancer Cell Lines

**Figure 2:**
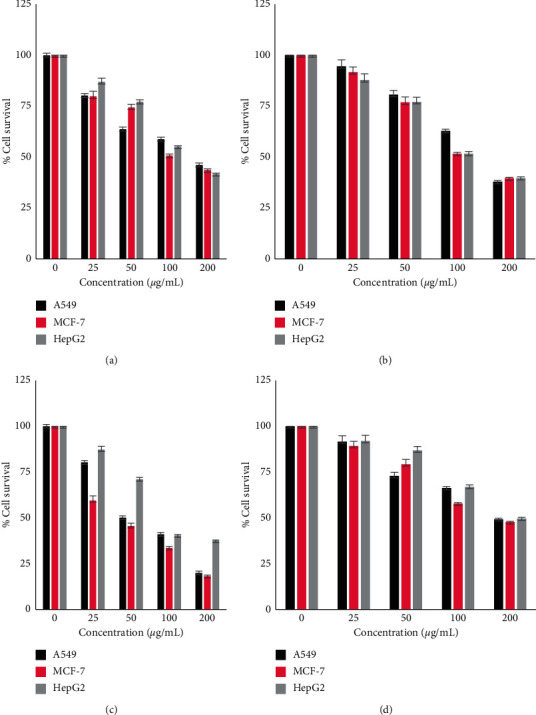
Inhibition of cellular viability of cancer cell lines by *S. glaucus* fractions. The cells were treated for 48 h and cytotoxicity of *S. glaucus* fractions was estimated using the MTT assay. Data represents the mean of three independent experiments, with each carried out in triplicate. (a) SG-crude. (b) SG-hex. (c) SG-CHCL_3_. (d) SG-BuOH.

All fractions showed cytotoxic activity in a dose-dependent manner (Figure 2). The most effective fractions were the chloroform fractions (IC_50_ = 41.8), which showed potent cytotoxic activity against MCF-7, followed by the hexane fraction, which showed moderate cytotoxicity in comparison with the remaining fractions. The results of the cytotoxicity evaluation in terms of IC_50_ against all tested cell lines are summarized in Table 2.

### 3.4. HPLC Analysis

**Figure 3:**
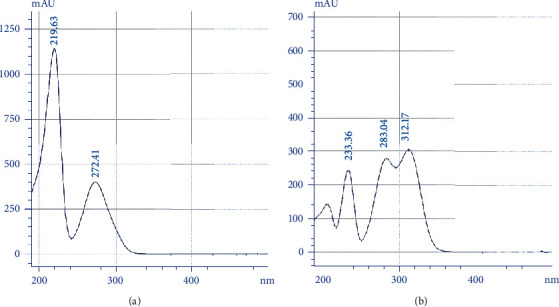
UV/vis spectrum scan of GA at a retention time of 10.89 min (a) and VA at a retention time of 14.45 min (b).

**Figure 4:**
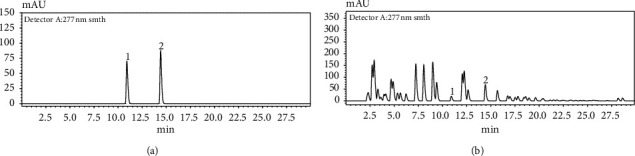
HPLC chromatograms of (a) standard phenolic acids and (b) extract. Peaks: (1) GA and (2) VA.

The wavelength maxima (*λ* max) of standard GA and VA were analyzed in order to obtain the optimal detection wavelength to be used for chromatographic analysis. The UV/vis spectrum scan of each HPLC peak was determined, and the detection wavelength was set according to the ultraviolet (UV) absorption maxima of the two analytes (Figure 3). Different mobile phase compositions, flow rates, and injection volumes were tested to improve the peak shape and resolution for the optimization of chromatographic conditions. Recognition and peak assignment of GA and VA were identified by comparing the mean of retention times and absorption spectrum of individual standards with the chromatographic profile of the extract (Figure 4). Once the optimum chromatographic conditions that allowed the satisfactory resolution of the two studied analytes were detected, the performance of the HPLC method was assessed by validating the method with an *S. glaucus* sample. The quality parameters of the chromatographic method, such as linearity, sensitivity, precision, and accuracy, were determined [25, 26].

### 3.5. Calibration Curves, Linearity, and Sensitivities (Detection Limits)

Detector response linearity was evaluated based on calibration curves. Calibration curves were studied based on the linear correlation between the peak area (*y*-axis) and concentration (*μ*g/mL) of the standard (*x*-axis). Linear curves for each analyte were constructed using six concentration points, in triplicate, for each phenolic compound. The correlation coefficient, intercept, and the slope of each calibration line were calculated using regression analysis. The linear range was found to be from 0.10 to 20.00 *μ*g/mL for GA and from 0.20 to 20.00 *μ*g/mL for VA. The values of the limit of detection (LOD) of the proposed method were 0.16 and 0.12 *μ*g/mL for GA and VA, respectively (Table 3), and those for the corresponding limit of quantification (LOQ) were 0.47 and 0.36 *μ*g/mL. The linear curves showed best-fit linear regression (coefficient of determination r^2^ > 0.997 for GA and VA, Table 3).

### 3.6. Precision and Accuracy

The quantification, repeatability (intraday), and intermediate precision (interday) of the method proposed here were measured and presented as the relative standard deviation (% RSD). The experimental data are shown in Table 4. The proposed method was precise, as indicated by % RSD values for the intraday (within 1 day) and interday (between three consecutive days) precision measurements. The precision assays for GA and VA were in accordance with the limit recommended by the International Conference on Harmonization (ICH) guidelines [27] as the % RSD values ranged from 1.13–3.45 to 1.53–4.10% for intra- and interassay precision values, respectively (Table 5).

Accuracy was determined through percent recovery with the addition of the standard solution to the *S. glaucus* sample at three different concentrations: 0.50 *μ*g/mL, 5.00 *μ*g/mL, and 20.00 *μ*g/mL. Table 5 shows the mean recoveries calculated on the basis of determination after exposing the samples with known amounts of analytes at three concentration levels. The mean recovery values of the two investigated analytes ranged from 95.43 to 100.39%, and their % RSD at each concentration was all less than 1.60%, which indicated that the proposed method was accurate enough for the quantification of VA and GA in *S. glaucus*.

## 4. Discussion

Plants are a valuable source of different phytochemicals that display various biological activities, including antioxidant and anticancer properties. The exploration of natural sources in search of biological agents with fewer side effects and good efficacy compared to synthesized compounds is continuously being conducted. In this report, we documented the profile of phenolic contents and the antioxidant and cytotoxic properties of *S. glaucus* found in Saudi Arabia. Species of the *Senecio* genus are known for containing an abundance of phenolic compounds [28–30]. Albayrak et al. (2014) reported that several species of the *Senecio* genus growing in Turkey contained high total phenolic contents that ranged from 11.63 ± 2.1 mg GAE/g extract in *S. viscosus* to 117.45 ± 1.8 mg GAE/g in *S. cilicius* [30]. The total phenolic contents for the same species found in Egypt also showed comparable values [19].

Interest in natural antioxidants from medicinal plants has increased due to the safety of these antioxidants in comparison with synthetic compounds [31]. These natural antioxidants, especially phenolic compounds, have a fundamental role in reducing the harmful effects of free radicals [32]. This correlation between phenolic compounds and scavenging activity was clearly reported in several studies [33–35]. The antioxidative potency of plants can be evaluated through their ability to scavenge free radicals. The DPPH and ABTS methods are the most widely utilized assays for determining free radical scavenging activity [36,37]. In this study, we showed that *S. glaucus* displayed strong antioxidant activity through two screening methods. The results were compared with a recent study which reported that essential oils from *S. glaucus* exerted a concentration-dependent DPPH scavenging activity [38]. Furthermore, Mohamed (2015) found that root methyl alcohol extract of *S. glaucus* growing in Egypt had an antioxidant activity with IC_50_ = 79.57 ± 0.74 *μ*g/ml using the DPPH method [19]. There were no major differences between our results and the results of other previously reported studies, and this may be due to the presence of the same chemical constituents, including GA and VA. Moreover, the presence of GA and VA, which have known antioxidant properties [27, 39], verified the reported antioxidant activity of *S. glaucus*. Furthermore, a hybrid of GA and another phenolic antioxidant strongly enhanced the plant's antioxidant potential [40].

Our results show that *S. glaucus* extracts had promising cytotoxic activity. We found that *S. glaucus* extracts decreased the viability of various cancer cells. Data from the literature on the cytotoxicity of *S. glaucus* are still scarce. A new study by Ramadan et al. (2020) showed that *S. glaucus* essential oils exerted cytotoxic activity against breast cancer cells [38]. Phenolic compounds, including GA and VA, are among the best candidates for mediating anticancer activities [7, 27, 39, 41]. It is apparent that phenolic contents determined by colorimetric standard methods do not give the exact quantity of the phenolic compounds found in plant extracts. Therefore, chromatographic methods, such as HPLC, play an important role in the isolation and quantification of phenolic compounds [42, 43]. Our study showed that GA and VA were abundant compounds in SGCF (Figure 3). These results are in agreement with Mohamed (2015), who detected the presence of GA, VA, and several other phenolic compounds from the roots of *S. glaucus* found in Egypt. Overall, these results demonstrate that the antioxidant and cytotoxic activities of *S. glaucus* were likely due to the combination of phenolic acids found in these species.

## 5. Conclusion

The present study determined the total phenolic contents of *S. glaucus* found in Saudi Arabia. In accordance with a recent study, our data indicate that *S. glaucus* are a good potential source of natural antioxidants, such as GA and VA. The chloroform fraction of *S. glaucus* (SGCF) exhibited the highest antioxidant and cytotoxic activities, which was positively correlated with their phenolic profile. The HPLC method we developed was approved and used to determine the GA and VA content in *S. glaucus*. It can be concluded that the combination of these two bioactive compounds in *S. glaucus* is crucial for the plants' antioxidant and cytotoxic activities which confirm their beneficial effect on human health. Thus, the findings of this study encourage further research in order to fully elucidate the bioactive constituents of *S. glaucus*, which may lead to the discovery of novel and effective drugs against degenerative diseases.

## Figures and Tables

**Table 1:** DPPH and ABTS scavenging activity of *S. glaucus* extract and fractions.

Sample	(DPPH radical scavenging activity in %)				
	10	50	100	500	1000
**SG-crude**	2.4 ± 3.4	10.4 ± 4.3	21.6 ± 3.6	35.9 ± 3.5	54.4 ± 2.7
**SG-hex**	2.3 ± 0.3	10.2 ± 3.1	19.7 ± 0.7	31.7 ± 2.1	44.7 ± 1.3
**SG-CHCl**_**3**_	12.7 ± 4.1	20.3 ± 4.3	43.3 ± 3.9	61.3 ± 4.1	76.7 ± 3.2
**SG-ButOH**	11.2 ± 3.6	19.1 ± 4.4	31.5 ± 3.4	47.5 ± 2	63. 6 ± 2.3
**Ascorbic acid**	80.7 ± 2.5	85.1 ± 1.3	85 ± 3.2	88.7 ± 2.4	90.7 ± 4.4

	(ABTS radical cation scavenging activity in %)				
**SG-crude**	6.21 ± 4.2	12.2 ± 3.1	22.3 ± 2.4	38.3 ± 4.6	51.6 ± 3.7
**SG-hex**	2.3 ± 3.1	5.4 ± 3.1	12.6 ± 2.7	20.6 ± 3.1	41.7 ± 3.1
**SG-CHCl**_**3**_	13.2 ± 3.3	19.3 ± 2.7	34.3 ± 3.2	56.3 ± 3.5	74.1 ± 2.3
**SG-ButOH**	6.21 ± 4.2	19.2 ± 3.1	32.3 ± 2.4	48.3 ± 4.6	61.6 ± 3.7
**Ascorbic acid**	80.7 ± 2.4	81.2 ± 2.1	84.2 ± 3.2	87.2 ± 2.4	88.7 ± 2.6

**Table 2:** The IC_50_ values of *S. glaucus* fractions in different cancer cells.

Fraction	IC_50_ values ± SD (*μ*g/mL)		
	MCF-7	HepG2	A549
Crude	101.3 ± 1.7	137.3 ± 1.1	170.1 ± 1.8
*n*-Hex	114 ± 1.5	112.3 ± 1.8	151.3 ± 1.6
CHCL_3_	41.8 ± 0.8	84.2 ± 1.2	49.8 ± 1.1
*n*-Butanol	178.6 ± 1.6	199 ± 1	196 ± 1.2
Doxorubicin	0.8 ± 0.2	1 ± 0.3	0.9 ± 0.4

**Table 3:** Linear regression parameters and sensitivity data for two phenolic acids using the proposed HPLC method.

Analyte	Retention time (min)	Range (*μ*g/mL)	Linearity (r^2^)	LOD (*μ*g/mL)	LOQ (*μ*g/mL)
GA	10.90 ± 0.03	0.10–20.00	0.9976	0.16	0.47
VA	14.42 ± 0.04	0.20–20.00	0.9989	0.12	0.36

**Table 4:** Analytical results of accuracy, intraday, and interday precision.

Analyte	Con. (*μ*g/mL)	Intraday (*n* = 3)		Interday (*n* = 3)	
		RSD (%)	Accuracy (%)	RSD (%)	Accuracy (%)
GA	0.5	1.94	99.57	3.44	97.54
	5	3.45	98.68	4.10	98.27
	20	2.61	97.77	1.53	98.26
VA	0.5	1.18	100.69	2.12	96.30
	5	2.87	98.10	2.32	98.71
	20	1.13	97.63	3.10	99.73

**Table 5:** Results of analytical recovery (*n* = 6).

Analyte	Added (*μ*g/mL)	Recovery (%) (mean ± SD)	RSD (%)
GA	0.5	98.02 ± 1.57	1.60
	5	99.01 ± 0.88	0.88
	20	95.43 ± 1.02	1.07
VA	0.5	97.93 ± 1.24	1.27
	5	96.07 ± 0.67	0.70
	20	100.39 ± 0.77	0.77

## Data Availability

The data sets used and/or analyzed during the current study are available from the corresponding author upon reasonable request.
